# Farmers’ Risk Preferences in Rural China: Measurements and Determinants

**DOI:** 10.3390/ijerph14070713

**Published:** 2017-06-30

**Authors:** Jianjun Jin, Rui He, Haozhou Gong, Xia Xu, Chunyang He

**Affiliations:** 1 State Key Laboratory of Earth Surface Processes and Resource Ecology, Beijing Normal University, Beijing 100875, China; xuxia02@126.com (X.X.); chunyanghe09@163.com (C.H.); 2 Faculty of Geographical Sciences, Beijing Normal University, Beijing 100875, China; ruihe23@126.com (R.H.); hzgong@163.com (H.G.)

**Keywords:** risk preference, farmer, survey question, risk experiment, China

## Abstract

This study measures farmers’ risk attitudes in rural China using a survey instrument and a complementary experiment conducted in the field with the same sample of subjects. Using a question asking people about their willingness to take risks “in general”, we found that the average response of our sample is slightly risk averse. Farmers’ exogenous factors (age, gender, and height) and self-reported happiness have a significant impact on farmers’ willingness to take risks. The experiment results show that approximately 44% of farmers in the study area are risk averse. We compare farmers’ self-reported measures of risk preferences derived from the survey instrument to preferences elicited through the experimental task. Results show that answers to the general risk attitude question in the survey can predict farmers’ behaviors in the experiment to a statistically significant degree. This paper can contribute to the empirical literature on comparing local farmers’ risk attitudes across different risk preference measurement methods in the developing world.

## 1. Introduction

Individual risk preferences are of fundamental importance for how people decide [1]. They have important impacts on outcomes in migration, technology adoption, savings, and risk-sharing among others [2,3,4]. Understanding individual risk preferences is a prerequisite to understanding economic behavior [5]. As a result, the literature on developing empirical measures of individual risk preferences has been growing, with the aim of capturing this important parameter of individual heterogeneity. However, individual willingness to bear risk is not straightforward to measure. Economists and psychologists have developed a variety of instruments in recent decades to elicit and assess individual risk preferences (for a review, see References [6,7]). 

Although there are many methods to elicit individual risk preferences, two broad approaches can be distinguished [8]. The first approach is to elicit risk preferences by asking individuals about personal traits directly related to risk aversion using survey questions (e.g., [9]). This method is relatively easy to use and cheap to administer. It usually needs a large sample and sometimes it is representative of the population. The issue related to this method, however, is that survey questions may not be incentive-compatible [10]. Various factors, including inattention, self-serving biases, and strategic motives could distort respondents’ risk preferences (for a discussion, see Reference [11]). Therefore, there is a need to validate respondents’ self-assessed risk preferences in a survey with other risk elicitation methods.

Experimental studies that measure risk-taking behavior with real money at stake can offer an incentive-compatible measure of risk preferences [12]. The experiment design can be controlled by researchers and needs fewer samples. A drawback of this technique is that it is costly and difficult to perform with a large sample [10]. There is a growing body of literature concerning the use of experimental data to elicit risk preferences in both developed and developing countries [13,14,15,16,17,18]. 

Both the survey method and the experimental method are widely used to measure an individual’s risk preference. Naturally, the question arises as to how risk preferences elicited by the two broad techniques compare to each other in a within-sample experiment. Thus, it is important to test the internal validity of the two alternative risk measurement mechanisms. In the last couple of years, there has been a growing body of literature analyzing how different methods to measure risk attitude can lead to different results [10,12,19,20,21,22,23]. However, a research gap persists in the comparison of the two different elicitation methods in China. One important objective of this study is to contribute to the literature to compare individuals’ risk preferences elicited by a survey instrument and a complementary experiment conducted in the field with the same sample of farmers in rural China. 

Furthermore, understanding the heterogeneity of farmers’ risk preferences is important, which can help policymakers to design more effective policies to help farmers to overcome several different potential sources of risk in developing countries [24]. However, there is no consensus on how risk preferences are influenced by socioeconomic characteristics. Although some studies find that risk preferences differ significantly based on gender (e.g., Reference [10]), age (e.g., Reference [17]), education (e.g., Reference [25]), and/or income (e.g., Reference [26]), others find no significant relationship for gender (e.g., Reference [25]), age (e.g., Reference [13]), or income (e.g., Reference [17]). Thus, the determinants of risk preference appear to be mixed and rather region-specific. More empirical research identifying the determinants of local farmers’ risk preferences are needed. The aim of this study is to look into the determinants of Chinese farmers’ risk preferences and to provide more empirical evidence to the literature.

The organization of this paper is as follows. Section 2 describes the survey risk measures that we use as well as our sample. Section 3 presents farmers’ self-assessment risk preferences in the survey and results from the complementary field experiment. Section 4 concludes the main findings of this paper and presents some policy implications.

## 2. Survey and Experimental Design

### 2.1. Survey Design

The survey questionnaire was finalized based on a series of focus group discussions and pre-tests. The questionnaire used for the survey included carefully designed questions to collect detailed information on respondents’ self-assessed risk preferences in general and the demographic and socioeconomic data of respondents and their households. For the general risk question, as in Reference [10], we directly asked respondents to give a judgment of their own willingness to take risks on a scale from 0 to 10, where 0 means: ‘not at all willing to take risks’ and 10 indicates: ‘very willing to take risks’. Respondents were also asked to report their happiness with their life on a scale from 0 to 10, where 0 indicates ‘completely unhappy’ and 10 stands for ‘completely happy’.

### 2.2. Design of the Experiment

The experiment consisted of a multiple price list (MPL) choice task developed based on the work by Brick et al. [27], which is variation on the widely-used Holt-Laury-type measure [13] and more akin to the approach by Eckel and Grossman [14]. In the choice task, subjects were shown different binary lotteries and they were asked to select either the “safe” option or the “risky” option that differed in terms of their associated risk and expected payoff. This design can be implemented with relative ease, and also encourages truthful revelation [28]. 

The choice tasks that were presented to subjects are reported in Table 1. In total, subjects were asked to compare eight option comparisons. As in Brick et al. [27], fixed probabilities of 100% and 50% were used. Under option A (the safe option), subjects can receive a certain amount of payoff for sure if they select this option. The payoff associated with this option declines systematically throughout the eight tasks. Option B (the risky option) has two payment levels (CNY 20 and CNY 0), which remain unchanged and are 50-50 likely to occur. Subjects were asked to indicate at which task they would “switch” from option A to option B. The difference in the expected values of the two options, which are replicated in the fourth column of Table 1, were not shown to subjects. As a risk-neutral subject would switch from option A to option B when the expected value of both is approximately the same [29], s/he would choose option A for the first three tasks and option B thereafter [27]. A risk-loving subject may choose option B in the second task, while a risk-averse subject may select option A in the eighth task. 

Based on this design, the switching point is informative about a subject’s risk attitude. We can obtain information about subjects’ degree of relative risk aversion by assuming that individuals have a constant relative risk aversion utility function: U(*x*) = *x*^1−r^/(1 − r), where *r* is the coefficient of relative risk aversion (CRRA), and *x* is the payoff in the option. Under this specification, a CRRA coefficient r > 0 implies risk aversion, r = 0 risk neutrality, and r < 0 risk seeking preferences. Column 5 in Table 1 provides the implied CRRA ranges.

### 2.3. Sample and Data Collection

Our study is based on a sample which is typical for the rural population in the central China. The study area was Yongqiao District, located in the north of Anhui Province. It is the biggest county-level district in China. The total area of this district is 2868 km^2^. In 2012, the population of the district was about 1,880,000, among which agricultural populations accounted for 78%. The average per capita Gross Domestic Product (GDP) was CNY 20,059 (USD 3280).

In order to obtain a representative sample, respondents were selected randomly from the villages in Yongqiao District. We first contacted the local village leaders to obtain some basic information and help to ensure the survey efficiency. We informed potential participants that if they participated in a study at the given location and time, they had an opportunity to earn cash. In most cases, household heads were invited. When the household head does mostly off-farm work (i.e., leave the village for working in big cities), we approached the family member who was the most responsible for farm work.

Each respondent was first asked to finish a questionnaire through face-to-face interviews. After completion of the questionnaire, respondents were asked to participate in the MPL experiment. The experiment was administered in a group setting, but choices were made individually and privately. For each group, approximately 10 persons participated. The group size was chosen consciously. Before commencing the experiments, subjects were provided with documentation detailing instructions and outlining the various option tasks. The instructions were read aloud and explained by well-trained experimenters. We did our best to help our subjects understand the instructions and subjects were encouraged to ask any questions related.

In order to ensure incentive-compatibility, we told subjects that they would receive a real payment from the experimenters, but that the amount would depend on the answers that they made in the experiment. Specifically, subjects were told that once they finish answering all tasks, one of these eight tasks would be selected at random and the choice they made in the selected task would be used to determine their payment. Each subject received a show-up fee (USD 6.5) to cover their expense for coming to the experiment.

The final survey was conducted in November 2013. A total of 220 household heads were invited and 200 persons finally attended our study. 

## 3. Empirical Results and Discussion

### 3.1. Demographic Profile of the Sample

The descriptive demographic profile of the valid sample is presented in Table 2. Approximately 46% of the sample was male, while the provincial average ratio of male was 51%. The typical respondent was 41 years of age. The overall average household size of the sampled respondents was five persons, while the provincial average was four. The average educational level was close to the middle school level (about nine years of education). Specifically, approximately 2% respondents were illiterate, and 22% had completed their primary education. Of the respondents, 58% and 18% had attained junior high school and higher education, respectively. The average household income was approximately 3467 Chinese Yuan per month (584 USD/month), which is fairly close to the provincial average of 3852 CNY/month.

### 3.2. Willingness to Take Risks in General

**Figure 1:**
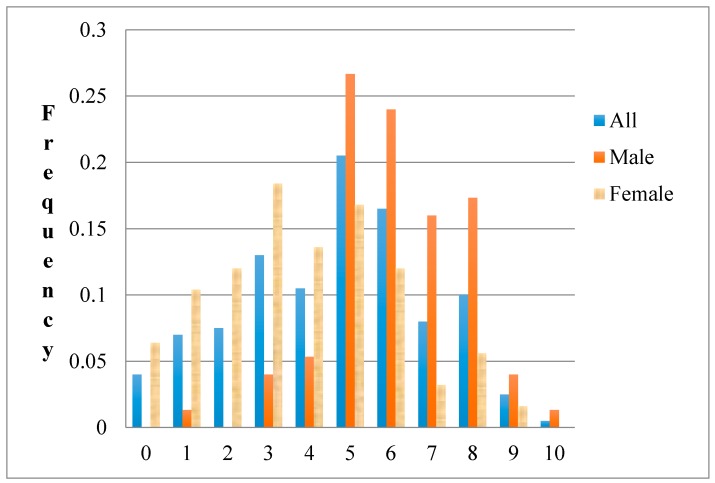
Histogram of responses to the question about willingness to take risks “in general”, measured on a 11-point scale (0 = not willing to take any risks; 10 = fully prepared to take risks).

In Figure 1, we describe the distribution of the whole sample, as well as men and women’s responses to the general risk question. The results reveal substantial heterogeneity in risk preferences across the sample. Roughly 8% of all individuals chose the extreme of 0, which indicates a complete unwillingness to take risks. A relatively small fraction of respondents (about 0.5%) chose a value of 10, indicating that they are very willing to take risks. The mean response was 4.68, indicating that the average response of our sample is slightly risk averse.

Figure 1 also shows men and women’s responses to the general risk question. It can be seen that the proportion of individuals who chose low values on the scale, that is, who are relatively unwilling to take risks, is higher for women. The percentage of men who chose high values on the scale is higher than the percentage of women.

In order to identify the determinants of these individual differences, we estimated ordered probit regressions where the dependent variable is the subject’s response to the general risk question [22]. Based on the findings of earlier literature [10,12,27], we include a number of independent variables in the model. First, the exogenous variables with respect to risk attitude were considered. This refers to the respondents’ gender and age, which are widely covered in the literature. In addition, some studies argue that tall people have physical advantages, are more disease-resistant, possess greater authority, and have better verbal and non-verbal abilities than do others [30,31,32]. Dohmen et al. [10] incorporate height as a control variable in their empirical risk function and find a significant positive effect. The ‘height’ variable cannot have a direct causal influence on risk behavior, but a statistical relationship is possible. Thus, as in Dohmen et al. [10], our baseline specification uses gender, age (years), and height (cm) as explanatory variables (see column 1). In the literature, the relation between risk preferences and individual self-reported assessments of happiness in life is particularly neglected. Previous studies have focused on how people’s satisfaction in life influences risky choices in certain contexts. For example, Goudie et al. [33] used American seatbelt data to demonstrate that happier people are less attracted to risk-taking. Column (2) shows the estimation results with an added indicator variable for respondents’ self-reported happiness level. We also include respondents’ socioeconomic variables (education levels, total monthly household income, and years in farming). The regression results are reported in Table 3.

The resulting coefficient estimates in column 1 show that men are significantly more willing to take risks in general. This finding is in line with the results in Figure 1. Willingness to take risks decreases with age significantly. Taller people are more willing to take risks. All of these effects are significant at the 1% level. These findings are consistent with the existing literature. For example, Dohmen et al. [10] found that taller people tend to accept more risk. Female and older people are more risk averse [19,34]. The results in column 2 reveal that the coefficient on happiness is positive and significant at the 1% level, indicating that people with high life satisfaction are more willing to take risks. The results shown in column 3 indicate that education has no effect on farmers’ willingness to take risks. The results in column 4 suggest that respondents’ household income level has no significant effect on their willingness to take risks. This finding is in line with the results of Brick et al. [10] and Mosley and Verschoor [35]. Our results in column 5 suggest that there is a positive and significant correlation between farmers’ farming experience and risk attitudes. 

### 3.3. MPL Results

Farmers’ risk preferences were first measured by the number of safe choices chosen in the experiment. The number of safe choices has a simple and natural interpretation, which allows us not to impose assumptions on the source or nature of risk preference noise. A greater number of safe options indicates a greater willingness to bear risk. The distribution of our subjects’ switching points is shown in Table 4. Closer investigation of farmers’ responses reveals that 18% of the sample exhibited risk-loving behavior. They switched earlier and chose fewer safe options. Approximately, the share of risk-neutral subjects was 39%, and 43% of the sample appeared to risk averse. This result is line with the one conducted by Brick et al. [10] in South Africa.

Using the midpoint of each CRRA class, the average CRRA coefficient in our experiment is estimated at 0.21, implying slightly risk aversion. This finding is comparable to the existing literature dealing with risk preferences of farmers using similar estimation methods. For example, Reynaud and Couture reported a mean CRRA coefficient for French farmers equal to 0.14 [5]; Brick et al. (South Africa) estimated the coefficient of CRRA to be 0.39 [10]; Qiu, Colson and Grebitus (USA) derived an estimate of 0.49 [36].

In order to explore the heterogeneity of subjects’ elicited risk preferences, we estimated an interval regression where the dependent variable is the CRRA interval. This regression model is not used to illustrate the causal relationship of subjects’ demographics on risk preferences; rather, it is used to look at the correlation between subjects’ demographics and risk preferences [36]. The estimation results are presented in Table 5.

The regression results indicate that gender is significantly negative at the 1% level, suggesting that women are more risk averse. The negative and significant coefficient on age suggests that older farmers are more risk averse. The coefficient for height is negative and significant, indicating that a taller person is more willing to take risk. People’s self-assessment on happiness, education levels, and household income have no significant effect on farmers’ risk preferences in the MPL experiment. However, farmers’ years in farming have a positive and significant coefficient, indicating that farmers with more farming experience are more risk averse.

### 3.4. Experiment and Self-Assessment

This section is to assess whether survey data can predict actual risk-taking behavior in the lottery experiment. We first compare individuals’ risk attitudes in the two methods. The results are presented in Table 6. In total, 38 subjects were consistently risk averse, which is about 62% of the risk-averse respondents in the survey method. 37 individuals (19% of the total sample) were considered risk neutral with both methods. 20 respondents (10% of the total sample) exhibited consistent risk-loving responses in the two methods. In total, approximately 48% were consistently considered as having the same risk attitudes with both methods.

We estimate various interval regression models in which the experimental outcome, that is, the choice of a switching point, is to be explained by the same person’s assessment in the general risk question. The dependent variable is the CRRA interval. Regression results are reported in Table 7.

In the first model, we simply regress the CRRA interval at the switching point on answers given to the general risk question. The coefficient on self-assessment risk attitude is positive and significant at the 1% level, indicating that the answers given in the survey do predict risk-taking behaviors in the experiment. This is of some economic importance. To check the robustness of this relation, we add controls in columns 2 and 3. Specifically, controls in column 2 include gender, age, and height, and in column 3 we control for additional individual characteristics, such as household income. The general risk coefficient stays significant. Therefore, the answers to the general risk attitude question in the questionnaire survey can be a proxy to predict their risk behaviors in the MPL experiment. One possible reason that can be used to explain this finding is that we use a non-standard pool of subjects made of Chinese farmers as our subjects. As Reynaud and Couture [5] point out, farmers are habituated in their professional life to take decisions in a context of uncertainty which may reduce the cognitive burden of the experimental task. Moreover, our sample is relatively homogenous in terms of income or education level.

## 4. Conclusions

A puzzling result that emerges from the literature on risk preference measure is that the risk preference of an individual may vary across elicitation techniques [5]. In this study, we employ a within-subject design to compare two different elicitation methods for measuring risk attitudes on a sample of non-standard subjects (Chinese farmers). Our study is unique in conducting a representative household survey with the subjects of an MPL experiment at the same time in a developing country [19].

The questionnaire survey measure asks people to give a judgement of their willingness to take risks in general. The field experiment employed an MPL choice design. We found an economically significant impact of gender, age, and height. Men are more willing to take risks than women. Increasing age leads to decreasing willing to take risks, and increasing height leads to a greater willingness to take risks. Happier individuals are more willing to take risks. 

The validity of the survey measure on the general risk is verified in the incentive-compatible field experiment. The results show that responses to the general risk question have a significantly positive impact on subjects’ decisions in the MPL experiment. This relation is not disturbed by considering several further possible determinants of risk attitudes. We confirm earlier findings in related literature [10,12,19]. Therefore, a well-designed survey item giving the self-assessed risk attitude of an individual may be a useful measure. However, some recent empirical evidence shows that individuals’ risk attitudes are context-dependent [37,38,39,40,41]. Even though the self-assessed general risk attitude predicts comparatively well, it may be well-advised to make these survey instruments context-specific, that is, geared to the problem at hand [42]. Moreover, our results show that only 48% of the total sample exhibited consistent risk attitudes in the two methods. This means that if researchers were going to use the survey method to predict attitudes/choices in real payment experiments, they have a 50% chance of being wrong. Finally, further research seems necessary to examine survey-experiment relations because individuals’ risk attitudes and their determinants may be country-specific or area-specific.

The findings of this study have some important implications for China and similar localities in other parts of the developing world. First, our results provide additional indications that female Chinese farmers are more risk averse than their male counterparts. This is in line with Croson and Gneezy’s review of the experimental literature relating to gender differences in risk behavior [43]. A gender difference in willingness to take risks could be part of the explanation for the important difference in social behavior and economic outcomes. An age profile for risk attitudes could also be important for explaining behavior at the macroeconomic level [10]. Our results also show that subjects who are satisfied with their life are more willing to take risks. Thus, it would be wise for policymakers to create an environment where optimism can flourish (e.g., good governance) to promote not only happiness in subjects but also to encourage them to be entrepreneurial. 

## Figures and Tables

**Table 1:** Experiment Design.

Task	Option A	Option B	EV ^A^–EV ^B^	CRRA Ranges
1	20 CNY	20 CNY if heads; 0 CNY if tails	10 CNY	r < −1.4
2	15 CNY	20 CNY if heads; 0 CNY if tails	5 CNY	−1.4 < r < −0.4
3	12 CNY	20 CNY if heads; 0 CNY if tails	2 CNY	−0.4 < r < 0
4	10 CNY	20 CNY if heads; 0 CNY if tails	0 CNY	0 < r < 0.2
5	8 CNY	20 CNY if heads; 0 CNY if tails	−2 CNY	0.2 < r < 0.4
6	6 CNY	20 CNY if heads; 0 CNY if tails	−4 CNY	0.4 < r < 0.6
7	4 CNY	20 CNY if heads; 0 CNY if tails	−6 CNY	0.6 < r < 0.7
8	2 CNY	20 CNY if heads; 0 CNY if tails	−8 CNY	0.7 < r

Notes: EV = Expected Value. CRRA = Coefficient of Relative Risk Aversion.

**Table 2:** Main socioeconomic variables and their mean values.

Variables	Description	Mean	Standard Deviation	Provincial Average
Gender	1 = male, 0 = female	0.46	0.49	0.51
Age	Age of respondent (years)	41	12	n/a
Height	Height of the respondent (cm)	163	6.65	n/a
Education	Education of the respondent (1 = No formal schooling, 2 = Elementary, 3 = Middle school, 4 = College, 5 = Masters or above)	2.96	0.80	2.50
Hhsize	Number of household members living together	4.80	1.30	4
Farmyears	Years in farming	19.72	13.18	n/a
Happiness	Self-reported happiness (0 = completely unhappy; 10 = completely happy)	7.78	2.14	n/a
Hincome	Household income (1000 CNY/month)	3.47	2.47	3.85

**Table 3:** Primary determinants of general risk attitudes.

Variable	(1)	(2)	(3)	(4)	(5)	(6)
Gender	0.63 *** (0.17)	0.63 *** (0.17)	0.64 *** (0.17)	0.63 *** (0.17)	0.64 *** (0.17)	0.65 *** (0.17)
Age	−0.03 *** (0.006)	−0.03 *** (0.006)	−0.03 ** (0.006)	−0.03 *** (0.007)	−0.05 *** (0.01)	−0.05 *** (0.01)
Height	0.05 *** (0.01)	0.05 *** (0.01)	0.06 *** (0.01)	0.05 *** (0.01)	0.05 *** (0.01)	0.05 *** (0.01)
Happiness		0.17 *** (0.04)				0.18 *** (0.37)
Eucation			−0.12 (0.07)			−0.08 (0.07)
Hincome				0.004 (0.03)		−0.02 (0.03)
Farmyears					0.02 ** (0.01)	0.02 * (0.01)
Log likelihood	−357	−346	−357	−356	−355	−343
LR Chi^2^	78.76 ***	100.69 ***	78.32 ***	78.79 ***	82.49 ***	106.78 ***
Observations	200	200	200	200	200	200

Notes: Standard errors are in parenthesis. ∗∗∗, ∗∗, ∗ indicate significance at the 1%, 5% and 10% level, respectively.

**Table 4:** Distribution of lottery choices in the experiment.

No. of Times Subject Choose Safe Option	Switch Point to the Lottery	No. of Subjects (%)
0	Always Lottery B	5 (2.5%)
1	2	5 (2.5%)
2	3	5 (2.5%)
3	4	20 (10%)
4	5	77 (38.5%)
5	6	50 (25%)
6	7	20 (10%)
7	8	6 (3%)
8	Always Lottery A	12 (6%)

**Table 5:** Multivariate correlates of the experiment.

Variable	Estimate	Std. Err.	*p*-Value	Lower 95% CI	Upper 95% CI
Gender	−0.27 ***	0.07	0.000	−0.41	−0.12
Age	0.02 ***	0.00	0.002	0.01	0.02
Height	−0.01 **	0.01	0.044	−0.02	0.00
Happiness	0.01	0.02	0.457	−0.02	0.04
Education	−0.04	0.03	0.276	−0.10	0.03
Hincome	0.00	0.00	0.141	−0.01	0.05
Farmyears	0.01 **	0.00	0.013	0.00	0.02
Constant	2.59 **	1.04	0.012	0.56	4.63
Summary statistics					
Log likelihood		−420			
LR Chi^2^		23.16 ***			
Observations		200			

Note: ∗∗∗ and ∗∗ indicate significance at the 5%, and 10% level, respectively. CI = Confidence Interval.

**Table 6:** Individuals’ risk preferences in MPL and self-assessment survey.

Category	MPL	Survey	Consistent Response
Risk aversion	86 (43%)	61 (31%)	38 (19%)
Risk neutral	77 (39%)	71 (36%)	37 (19%)
Risk lover	37 (18%)	68 (34%)	20 (10%)

**Table 7:** Multivariate correlates of the experiment and survey measures.

Variables	(1)	(2)	(3)
Constant	−0.09 (0.08)	2.76 *** (1.04)	2.82 *** (1.04)
General willingness to take risk	0.11 *** (0.05)	0.04 ** (0.02)	0.03 * (0.02)
Gender		−0.21 *** (0.08)	−0.23 * (0.08)
Age		−0.003 (0.001)	0.01 ** (0.004)
Height		−0.02 *** (0.006)	−0.01 ** (0.06)
Happiness			0.001 (0.02)
Education			–0.03 (0.03)
Hincome			0.02 (0.01)
Farmyears			0.01** (0.004)
Log sigma	0.76 *** (0.05)	0.79 *** (0.05)	0.81 *** (0.05)
Log likelihood	−428	−423	−418
LR Chi^2^	7.03 ***	17.02 ***	25.93 ***
Observations	200	200	200

Notes: Standard errors are in parenthesis. ***, **, * indicate significant at the 1%, 5%, and 10% levels, respectively.
